# Exploring the Expanding Role of Nuclear Receptor LRH‐1/NR5A2 in Cell Biology and Immunity

**DOI:** 10.1155/ijcb/3335387

**Published:** 2026-10-06

**Authors:** Eleni Tsapalou, Panagiotis Michael, Maria Georgoula, Konstantinos Michail, Stamatia Papoutsopoulou, Barry J. Campbell

**Affiliations:** ^1^ Department of Biochemistry and Biotechnology School of Health Sciences University of Thessaly Biopolis Campus Larissa Greece uth.gr; ^2^ Department of Molecular Biology and Genetics Democritus University of Thrace Alexandroupolis Greece duth.gr; ^3^ Department of Infection Biology and Microbiomes Institute of Infection Veterinary and Ecological Sciences University of Liverpool Liverpool UK liv.ac.uk; ^4^ Faculty of Medicine University of Thessaly Biopolis Campus Larissa Greece uth.gr

**Keywords:** epithelium, immune cells, liver receptor homolog-1, nuclear receptor subfamily 5 group A member 2, orphan receptor, transcription factor

## Abstract

LRH‐1/NR5A2 is a member of the NR5A subfamily of nuclear receptors, acting as a crucial transcription factor involved in diverse biological processes. Although LRH‐1 has been extensively studied in vitro and in vivo, in relation to its role in embryonic development and differentiation and in adult tissue processes, such as cholesterol, glucose, and ethanol metabolism, current knowledge about its function in immunity is very limited. This review, therefore, presents the most recent knowledge regarding *NR5A2* regulation and LRH‐1 function, including the wide spectrum of its target genes. In addition to LRH‐1 regulating steroidogenesis in tissues such as the ovaries, testes, pancreas, and breast adipocytes, it is also key in driving glucocorticoid production in intestine, where its immunomodulatory activity is undoubtedly important in maintaining gut homeostasis and countering inflammation. Likewise, LRH‐1 also appears to be crucial to the enterohepatic circulation, regulating hepatic bile acid synthesis and secretion, and intestinal transport/reabsorption. Our review also highlights fundamental roles for LRH‐1 in macrophage polarization and in T cell homeostasis, revealing a major role in Th2 immune responses. Although no studies to date have explored the association of LRH‐1 with respect to B‐lymphocyte biology, we highlight the need for understanding its wider role in the immune system and its interplay with tissues and organs in health and disease.

## 1. Introduction

Liver receptor homolog‐1 (LRH‐1), also referred to as nuclear receptor subfamily 5 group A member 2 (NR5A2), is one of four members of the NR5A family of nuclear receptors [1]. LRH‐1 acts as a transcription factor, where its nuclear expression (Figure 1A) is critical for regulating a wide range of physiological processes, with its activity modulated by several key coactivators, corepressors, and posttranslational modifications, which can lead to tissue‐specific functions. LRH‐1/NR5A2 is also known as *α*1‐fetoprotein transcription factor (FTF) [3], CYP7A promoter‐binding factor (CPF), human B1‐binding factor (hB1F), pancreas homolog receptor‐1 (PHR‐1), and is the mammalian homolog of the *Drosophila* fushi tarazu F1 (FTZ‐F1; Nr5a3). The human *NR5A2*/*hB1F* gene was originally cloned in 1998, based on its interaction with hepatitis B virus (HBV) enhancer II B1 element, thus regulating expression of the HBV open reading frames in liver [1, 3, 4]. Five years later, the first transgenic mouse expressing LRH‐1 was made to study the genes and pathways that are regulated by this protein [5]. *NR5A2* maps to chromosome 1q32.11 [1] and encompasses eight exons that can result in different mRNAs via alternative splicing (Figure 1B). The largest hLRH‐1 variant is Isoform 1, and the most prevalent is Isoform 2, which closely resembles Isoform 1 except for a missing segment in the A/B domain corresponding to Exon 2 [6]. The smallest variant Isoform 3 has an additional deletion affecting the D and E domains due to alternative splicing in Exon 5, rendering it unable to activate transcription [7, 8]. The current knowledge of human LRH‐1 isoform function is largely restricted to Isoforms 2 and 1, with the physiological significance of Isoform 3 relatively unexplored. These isoforms play distinct roles in disease states like cancer, where increased transcriptional activation of *β*‐catenin (a key nuclear effector in the Wingless [Wnt] signaling pathway) and its downstream targets such as c‐Myc (a master regulator of cell metabolism and proliferation) has been associated with the overexpression of LRH‐1 isoforms, contributing to gastrointestinal tumorigenesis [6]. Nadolny and Dong [6] documented that human gastric adenocarcinoma is characterized by the coexpression and upregulation of Isoforms 1 and 2. Murine Lrh‐1, encoded by the *Lrh-1*/*Nr5a2* gene, also gives rise to multiple isoforms important in either the developmental stages of embryogenesis and/or adult tissues [9].

**Figure 1:**
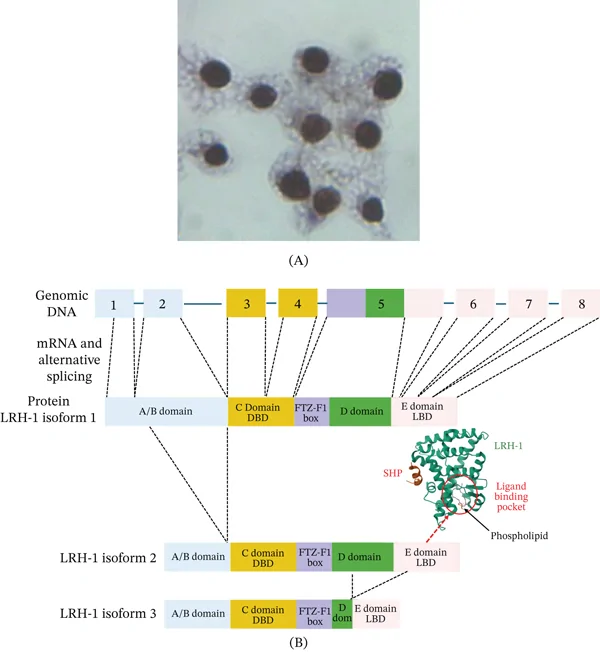
The nuclear receptor LRH‐1. (A) Immunocytochemistry showing LRH‐1 nuclear staining in resting J774A.1 murine macrophages (P.M. and B.J.C., unpublished data, using an antimouse Lrh‐1 antibody). (B) Human *LRH-1*/*NR5A2* genomic locus organization and LRH‐1 protein Isoforms 1–3, showing all structural domains; the N‐terminal A/B domain harboring a ligand‐independent activation motif crucial to receptor function; the C domain (or DBD); the FTZ‐F1 box; the D domain, which underdoes posttranslational modifications that modulate transcriptional activity; and connects the DBD to the E domain (the LBD) that facilitates interactions with coactivators and corepressors that regulate transcription. The LBD consists of 12 *α*‐helical regions and contains a ligand‐binding pocket capable of interacting with molecules such as phospholipids. 3D structural image from the RCSB Protein Data Bank (PDB); PDB ID: 1YUC (http://www.rcsb.org/structure/1YUC). Illustrated is the human nuclear receptor LRH‐1 (hLRH‐1/NR5A2) bound to a phospholipid ligand and a fragment of LRH‐1 corepressor SHP (small heterodimer partner; PDB doi:10.2210/pdb1YUC/pdb) [2].

Being part of the nuclear receptor superfamily, LRH‐1 protein follows the conserved modular structure of these receptors while exhibiting some distinctive features. Specifically, the LRH‐1 protein comprises four key structural domains [1, 10]; see Figure 1B. The N‐terminal A/B domain contains a ligand‐independent activation motif, activation function‐1 (AF‐1), that plays a crucial role in receptor function. The C‐domain or DNA binding domain (DBD) is highly conserved and consists of two zinc finger motifs. The FTZ‐F1 box, a 30‐amino acid extension, is located at the C‐terminus of the DBD. The fourth E domain, or ligand‐binding domain (LBD), includes the conserved activation function‐2 (AF‐2) motif, which is ligand‐dependent and facilitates interactions with coactivators to regulate transcription. The LBD consists of 12 *α*‐helical regions, numbered H1–H12, which are folded into a three‐layered, antiparallel helical sandwich with a conserved *β*‐turn between H5 and H6. The LBD also contains a ligand‐binding pocket capable of interacting with molecules like phospholipids [10]. Finally, the D domain connects the DBD to the LBD and undergoes posttranslational modifications that modulate transcriptional activity [7, 11, 12].

LRH‐1/NR5A2 is primarily expressed in enterohepatic tissues (liver and intestine), with additional expression seen in other organs, including the pancreas, ovaries, and placenta [13, 14]. Single blastomere RNA sequencing has recently revealed an NR5A2‐associated metabolic transcriptional signature in porcine embryos [15], while its role in adipose tissue has also been explored [16]. LRH‐1/NR5A2 expression is also detectable in immune cells, such as macrophages (Figure 1A) and T lymphocytes [17]. This diverse expression implies that this key nuclear receptor/transcription factor is involved in regulation of a variety of biological processes. In this review, we present the current knowledge about the function and regulation of LRH‐1/NR5A2, including focus on the immune system where its role is relatively less explored.

## 2. Regulation of LRH‐1/NR5A2 Expression

The expression of *LRH-1*/*NR5A2* has been studied in several species, mainly due to its function as a developmental regulator [18]. The variety of transcription factors that control its transcription, in a tissue‐specific manner, and the additional epigenetic regulators, such as microRNAs, that affect its expression, will be discussed below.

### 2.1. Transcriptional Regulation

The complete genomic sequence of human *NR5A2* was revealed in 2001 by Zhang et al. [8]. In that study, a bacterial artificial chromosome (BAC) library was screened, and binding sites for several ubiquitous and liver‐specific transcription factors were identified, including the hepatocyte nuclear factors HNF1 and HNF3*β* that could induce the expression of *NR5A2* [8]. Hepatocyte‐specific activation was also shown for the murine *Nr5a2* (*FTF*) promoter [19]. Functional analysis of the murine locus showed that it is regulated by GATA, HNF4*α*, FTF, Nkx, and basic helix‐loop‐helix factors in cell lines that express endogenous LRH‐1, such as HepG2 hepatocytes [19].

Bile acids can also induce *NR5A2* expression in HepG2 cells and in liver tissue from experimental rats, probably due to farnesoid X receptor (FXR) activity [20]. Nuclear factor kappa B (NF‐*κ*B) transcription factor was shown by chromatin immunoprecipitation (ChIP) assays to be a negative regulator of LRH‐1 expression in mouse liver homogenates [21]. Annicotte et al. [22] showed that LRH‐1 and pancreatic‐duodenal homeobox 1 (PDX‐1) are coexpressed in the pancreas during mouse embryonic development. In vitro and in vivo experiments during pancreatic development revealed that PDX‐1 directly regulates the expression of LRH‐1 [22]. LRH‐1 expression has been detected in the ovaries [23]. Cloning and mRNA expression analysis of the equine *NR5A2* gene revealed that it is mainly expressed in granulosa cells [23]. When primary ovarian cells were treated with human chorionic gonadotropin (CG), an inducer of ovulation, downregulation of *NR5A2* mRNA levels was observed [23]. Transgenic mice expressing *lacZ* under the control of the FTF‐promoter showed that LRH‐1 is selectively expressed in granulosa cells of the ovaries and is regulated/induced by follicle‐stimulating hormone (FSH) [24]. FSH was later shown to induce *NR5A2* expression in primary rat granulosa cells via activation of CREB (cyclic AMP response element‐binding transcription factor) and CRTC2 (CREB‐regulated transcription coactivator 2) [25]. In ovine ovarian cells, a specific mutation in the *Nr5a2* promoter created a novel binding site for metal‐regulatory transcription factor 1 (Mtf‐1), induced Lrh‐1 expression leading to a higher rate of reproduction, and more offspring in Hu sheep [26]. Conversely, in prostate cancer, where androgen receptor (AR) signaling is a key disease driver and primary therapeutic target, androgen‐activated AR appears to be a negative regulator of LRH‐1/NR5A2, whereas antiandrogen therapies can relieve this suppression through upregulating nuclear receptor expression [27]. Using *Ctnnb1* ^−/−^ and *Lrh-1* ^−/−^ embryonic stem cells, Wagner et al. [28] showed that *Lrh-1* is a novel direct target gene of *β*‐catenin and Tcf3 that regulates gene expression maintaining pluripotency. *NR5A2* silencing was also shown to inhibit the proliferation and migration of pancreatic cancer cells in vitro and in vivo, but its expression was induced by bromodomain‐containing protein 4 (BRD4) [29]. In human colonic crypt stem cell‐derived organoid cultures or CCD841CoN cells (an adherent cell line isolated from colon tissue of a healthy donor), glucocorticoid receptor (GR) can directly activate *NR5A2* transcription, contributing to intestinal steroidogenesis [30]. More recently, Guo et al. [31] showed that deoxynivalenol, one of the most detected mycotoxins in cereals, is a potent inducer of *Nr5a2* gene in vivo, modulating the oxidative stress/damage response genes *Ncoa4* and *Prdx*3 that encode ferritin cargo receptor NCOA4 and peroxiredoxin 3, respectively, in murine intestinal epithelium.

### 2.2. Epigenetic Regulation

Dysregulation of *LRH-1*/*NR5A2* has been linked to pancreatic, breast, and gastrointestinal cancers, where it has been shown to promote cell proliferation and metastasis [6]. Noncoding RNAs appear to play an important role, where multiple microRNAs bind the 3 ^′^ UTR of the *NR5A2* mRNA, acting as negative regulators. These microRNAs are downregulated in cancer leading to increased LRH‐1 levels [6]. In colorectal carcinoma, miR‐30d‐5p (formerly known as miR‐30d) is downregulated [32]. Its overexpression leads to suppression of tumor progression by directly targeting and downregulating LRH‐1 expression [32, 33]. Similarly, overexpression of miR‐381 [34], miR‐136 [35], and miR‐374b [36] inhibited tumor progression, via the Wnt/LRH‐1 pathway, in colon cancer. In patient tissues with nonsmall cell lung cancer (NSCLC), there is a strong negative correlation between the levels of miR‐376c and LRH‐1, and overexpression of miR‐376c in NSCLC cell‐line cultures limits cancer growth and invasion by decreasing LRH‐1 and through interference of Wnt pathway signaling [37]. Similar observations have been noted for miR‐381 in hepatocellular carcinoma (HCC) [38] and miR‐433‐3p in glioma, both acting through suppression of *NR5A2* [39]. The microRNA miR‐219‐5p negatively regulates LRH‐1 expression by binding to the 3 ^′^‐UTR of *NR5A2* mRNA. Upregulation of miR‐219‐5p has been observed in various other conditions, such as cyanotic congenital heart disease patients [40], in mouse models of spinal cord injury [41] and in diabetic retinopathy, as seen in human retinal pigment epithelial cells treated with high levels of glucose [42]. The increased miR‐219‐5p levels can interfere with Wnt/LRH‐1 signaling, limit LRH‐1 levels and its protective effects and lead to cell death. By blocking miR‐219‐5p, Li et al. [41] managed to restore LRH‐1/Wnt signaling, leading to neuronal protection. On the other hand, the downregulation of miR‐219‐5p is associated with gastric cancer, whereas miR‐219‐5p overexpression suppressed LRH‐1 levels and reduced cancer cell proliferation, migration, and invasion [43]. In a mouse model of intestinal‐failure–associated liver disease, probiotic microbiota metabolite (indole‐3‐acetic acid) driven suppression of the microRNA miR‐216a‐5p led to LRH‐1 activation and protection of hepatocytes from injury [44]. This study highlights that approaches targeting LRH‐1 could alleviate diabetes‐induced lipotoxicity in the liver (and other tissues), where cellular damage results from ectopic accumulation of lipids. MicroRNA miR‐139‐5p, another negative regulator of LRH‐1, is downregulated in esophageal squamous cell carcinoma and its overexpression could suppress cell proliferation, migration, invasion, and induction of apoptosis [45]. However, unlike other microRNAs, in glioblastoma multiforme the miR‐139‐5p levels are negatively regulated by the long noncoding RNA (lncRNA) RP3‐439F8.1, where it is part of a broader regulatory network [46]. In that study, RP3‐439F8.1 was shown to be a positive regulator of *LRH-1* and enhances LRH‐1 activity by reducing miR‐139‐5p levels [46]. Understanding the epigenetic regulatory network of LRH‐1 within various types of cells is important, as tailoring treatments to the unique regulatory dynamics of each condition may unlock new avenues for targeted and effective therapies.

## 3. Regulation of LRH‐1 Function

LRH‐1 was initially considered an orphan nuclear receptor because there were not any known endogenous ligands; however, natural phospholipids and synthetic lipophilic chemical ligands have been identified [7]. Additionally, LRH‐1 activity can be directly modulated by coregulators (both coactivators and corepressors), agonists, inverse agonists, and antagonists, binding to the *α*‐helical region H12 in the LBD and leading to conformational changes of LRH‐1 [10, 47], [48]. Moreover, indirect regulation can be achieved via posttranslational modifications, such as sumoylation and phosphorylation [49–51].

### 3.1. Natural Ligands and Synthetic Agonists

The activity of LRH‐1 can be modulated by phosphatidylcholines [52–54], phosphatidylethanolamines [25], and phosphatidylinositol phosphates, such as phosphatidylinositol 4,5‐bisphosphate [PI(4,5)P2 or PIP2] and phosphatidylinositol (3,4,5)‐trisphosphate [PI(3,4,5)P3 or PIP3] [47, 53]. Synthetic phosphatidylcholines 1,2‐dilauroyl‐sn‐glycero‐3‐phosphocholine (DLPC) and 1,2‐diundecanoyl‐sn‐glycero‐3‐phosphocholine (DUPC), distinguished by their short fatty acid chains (12 carbons for DLPC and 11 for DUPC) and considered as natural ligands, also bind to LRH‐1 and have potent agonist activity [52, 55]. A recent study has identified bilirubin, the product of liver heme metabolism, as a natural ligand of LRH‐1 in silico, in vitro, and in HEK293T cell culture bioassays [56].

Whitby et al. [57] reported a small LRH‐1 agonist, GSK8470, that effectively induced the transcriptional activation of steroidogenic genes. The same group optimized the structure of GSK8470 and synthesized new agonists, RJW100‐RJW103 [58]. Later, a study based on x‐ray crystallography showed that the two closely related agonists with similar efficacy, GSK8470 and RJW100, exhibit completely different binding modes, resulting in distinct mechanisms of action [59]. The same research group designed novel phospholipid mimics that were much more effective agonists of LRH‐1, such as 6HP‐CN [60]. These structure‐guided approaches led to the production of the synthetic agonist 6N that could upregulate steroidogenic gene expression in an in vitro organoid model [61–63]. Later, another 6N‐10CA compound was developed that had increased affinity and potency when compared with RJW100, 6N, or 10CA alone. Another small agonist, BL001, was described by Cobo‐Vuilleumier et al. [64]. BL001 could activate LRH‐1 in vitro and aided the functionality of pancreatic endocrine cells, reducing the apoptosis of *β* cells in the pancreatic islets of Type 2 diabetic mice [64].

A recent fluorescence resonance energy transfer (FRET)–based screening study identified and verified that the pesticide abamectin, a mixture of two closely related macrocyclic lactones, interacts with the full‐length LRH‐1 in cells [65]. More recently, Lang et al. [66] tested and showed that three derivatives of venlafaxine, a tertiary amino alcohol with aromatic and cyclic components and used as an antidepressant drug, can be LRH‐1 agonists, with these derivatives showing improved activation efficacy and selectivity over the parent compound.

### 3.2. Inverse Agonists and Antagonists

Busby et al. [67] selected the synthetic inverse agonists of LRH‐1, ML179 (SR‐1309), and ML180 (SR‐1848) for studies in cancer, where both compounds were able to inhibit cancer cell growth in vitro. SR‐1848 was also shown to reduce cell proliferation of many different cell types and inhibit aromatase activity, which could be a potential target against breast cancer [68].

In 2013, Benod et al. [11] screened 5.2 million compounds by molecular docking and identified compounds Cpd3 and Cpd3d2 as potential small‐molecule antagonists of the nuclear receptor LRH‐1/NR5A2, which then were evaluated in vitro [69]. Cpd3 and Cpd3d2 were effective synthetic inhibitors of LRH‐1 transcriptional activity of genes related to breast, pancreatic and colon cancer cells [11]. Cpd3 has been used in vitro in primary human pancreatic ductal adenocarcinoma cells with successful inhibition of LRH‐1 activity without any observable cell toxicity [70]. Additionally, experiments in Zebrafish (*Danio rerio*) showed that Cpd3 inhibits LRH‐1 and consequently affects hepatopancreatic development [71]. More recently, structure‐based design strategies have identified and characterized a novel class of indole‐based LRH‐1 antagonists with therapeutic potential, where compounds XY25026 and XY25028 demonstrate significant antitumor activity in a 22Rv1 xenograft model of androgen responsive prostate cancer [72].

### 3.3. Coactivators and Corepressors

Common coactivators of LRH‐1 include members of the p160 family, like steroid receptor coactivators SRC‐1, SRC‐2, and SRC‐3, as well as transcriptional coactivator p300. Interactions between LRH‐1 and the SRC coactivators are facilitated through the LXXLL motif of their receptor interaction region and the AF‐2 region of LRH‐1 in liver epithelial‐like Huh 7 cells and HepG2 cells [73]. The SRC‐1 protein has been implicated in cholesterol synthesis [74], and SRC‐3 has been shown to be involved in steroid hormone signaling [73]; however, the role of the SRCs in colorectal cancer is not clear [75]. The multiprotein bridging factor (MBF‐1) is also a coactivator of LRH‐1, acting to bridge it with a multisubunit complex known as transcription factor IID (TFIID) [76]. Additionally, peroxisome proliferator‐activated receptor gamma coactivator 1‐alpha (PGC‐1*α*) interacts with LRH‐1, probably promoting conformational changes that aid in agonist binding, and hence transcriptional activation [59]. In hepatocytes, PGC‐1*α* is identified to be involved in the expression of cholesterol 7*α*‐hydroxylase (CYP7A1) that catalyzes the rate‐limiting step in the classic pathway of hepatic bile acid biosynthesis from cholesterol [77]. PGC‐1*α* and LRH‐1 were shown to regulate aromatase gene expression in primary human breast preadipocytes [78]. Lastly, *β*‐catenin, implicated in cell proliferation and cell cycle advancement, can synergize with LRH‐1 to express cyclin E1 within crypt cells of the intestine [79, 80].

Negative regulation of the LRH‐1 activity can be achieved through the engagement of corepressors. The silencing mediator for retinoic acid receptor and thyroid hormone receptor (SMRT) has been shown to inhibit LRH‐1 transcriptional activity, most likely through indirect mechanisms rather than through any direct interaction [81]. Two members of the nuclear receptor subfamily 0 group B member 2 (NR0B) have been identified as effective corepressors of LRH‐1, dosage‐sensitive sex reversal, adrenal hypoplasia critical region, on chromosome X, gene 1 (DAX‐1/NR0B1), and small heterodimer partner (SHP/NR0B2), where in both proteins, the LXXLL motif mediates the interaction with LRH‐1 [82]. DAX‐1 and LRH‐1 are both expressed in granulosa cells and successful inhibition of LRH‐1‐mediated transcription of *3HSD2*, a gene that encodes a bifunctional enzyme crucial for the biosynthesis of all classes of hormonal steroids in those cells, has been reported [82]. LRH‐1 enhances the expression of SHP that, in turn, reduces the transcriptional activity of LRH‐1, creating a negative feedback loop [83]. Negative regulation from SHP can also be achieved by competition with agonists for binding in the LBD [2, 73].

The known regulators that have been reported so far for LRH‐1 are summarized within Table 1.

**Table 1:** LRH‐1 natural ligands, synthetic ligands, and coregulators.

**Ligands**			

**Natural**	**Synthetic**		
	***Agonists***	***Inverse agonists***	***Antagonists***

Bilirubin, DLPC DUPC PC PE PIP2, PIP3	6HP‐CN, 6N 6N‐10CA, 10CA Abamectin, BL‐001 GSK8470, RJW100, RJW101 RJW102, RJW103 Venlafaxine‐derivatives	ML179 (SR‐1309) ML180 (SR‐1848)	Cpd3, Cpd3d2 Indole‐based derivatives XY25026, XY25028

**Coregulators**			

**Coactivators**	**Corepressors**		

*β*‐catenin MBF‐1 p300 PGC‐1*α* SRC‐1, SRC‐2, SRC‐3	DAX‐1		
	SHP		
	SMRT		





Abbreviations: DAX‐1, dosage‐sensitive sex reversal, adrenal hypoplasia critical region, on chromosome X, gene 1; DLPC, 1,2‐dilauroyl‐sn‐glycero‐3‐phosphocholine; DUPC, 1,2‐diundecanoyl‐sn‐glycero‐3‐phosphocholine; MBF‐1, multiprotein bridging factor 1; PC, phosphatidylcholines; PE, phosphatidylethanolamines; PGC‐1*α*, peroxisome proliferator‐activated receptor gamma coactivator 1‐alpha; PIP2, phosphatidylinositol 4,5‐bisphosphate [PI(4,5)P2]; PIP3, phosphatidylinositol (3,4,5)‐trisphosphate [PI(3,4,5)P3]; small heterodimer partner; SMRT, silencing mediator for retinoic acid receptor and thyroid hormone receptor; SRC, steroid receptor coactivator.

### 3.4. Posttranslational Modifications

Among the known posttranslational modifications, phosphorylation and sumoylation have been detected so far for LRH‐1 protein [84]. Phosphorylation leads to LRH‐1 activation [85]. Extracellular signal‐regulated kinases can phosphorylate serine residues S238 and S243 of the hinge, and protein kinase A can phosphorylate residue S496 to increase biological activity of LRH‐1 [85]. Sumoylation acts as a repressor of LRH‐1 activity by aiding the sustainment of corepressors bound with LRH‐1, and in that way, decreasing LRH‐1 activity. Major sumoylation sites include lysine residues K192 and K270, and to a lesser extent, K44 [86–88].

## 4. Target Genes and Biological Role of LRH‐1

LRH‐1 is shown to regulate early development [5, 89, 90], glucocorticoid production in intestine [91], pluripotency and somatic cell reprogramming [28, 92, 93], bile acid homeostasis [94], ethanol metabolism [95], cholesterol metabolism, and glucose homeostasis [96–98]. A comprehensive list of LRH‐1 target genes is presented in Table 2.

**Table 2:** LRH‐1/NR5A2‐dependent target genes and biological roles in murine and human studies.

Biological roles	Target genes	References
Oxidative stress	*Ncoa4* and *Prdx*3	[31]
Hepatitis B proliferation	*ENII* of *HBV* virus	[4]
Pluripotency and development	*Gata6*, *Klf5*, *LIN28B*, *NANOG/Nanog*, *OCT4*/*Oct4*, *SOX2*, *Tbx3*, and *Xist*	[15, 18, 28, 99]
Steroidogenesis	*ABCB11*, *ABCG5*, *ABCG8*, *CYP7A1/Cyp7a1*, *CYP8b1/Cyp8b1*, *CYP11A1*, *CYP11B1*, *CYP11B2*, *CYP19*, *CYP17*, *CYP27A1*, *Greb1*, *HSD3B1*, *HSD3B2*, *SCARB1*, *SHP*, *StAR*, and *Tff1*	[52, 91, 94, 100–106]
Liver, lipid, and bile acid homeostasis	*ABCB11*, *ACC2*, *CYP7A1*, *FABP*, *CYP8B1*, *CPT1B*, *Elovl5*, *Gls2*, *Got1*, *Gpt2*, *LPL*, *Ostα*, *Ostβ*, *Pp1r3c*, *SCARB1*, and *VLDLR*	[105, 107–113]
Glucose homeostasis	*Acc2*, *Cpt1b*, *Fabp3*, *Gck*, *Glut4*, *Lpl*, and *Vldlr*	[113, 114]
Alcohol metabolism	*ALDH1B1*	[95]
Cell renewal, proliferation, and transition	*CCND1*, *CCNE1/Ccne1*, *CDKN1A*, *c-myc*, *MMP25/Mmp25*, and *Notch*	[70, 80, 115–117]
Pancreas function	*CEL/Cel*, *Cpa1*, *Ctrl*, *Ela*, *Pnlip*, *Rbpjl*, and *Shp*	[118, 119]
Fas/CD95 ligand (FasL)‐induced cell death	*FASLG*	[120]

### 4.1. Pluripotency and Early Development

LRH‐1 serves an essential function in early development and differentiation, as demonstrated by the embryonic lethality of homozygous *Lrh-1*/*Nr5a2* knockout mice [94, 121]. During the epiblast stage of embryogenesis, LRH‐1 critically maintains octamer‐binding transcription factor 4 (*Oct4*) gene expression, thereby ensuring pluripotency in embryonic stem cells [28, 122]. Using *Lrh1* ^−/−^ embryonic stem cells, Wagner et al. [28] showed that LRH‐1 regulates the transcription of pluripotency and development regulators such as the transcription factors Oct4/POU5F1 (*Oct4*), homeobox protein Nanog (*Nanog*), and T‐Box Transcription Factor 3 (*Tbx3*). Notably, LRH‐1 can effectively replace Oct4 in the reprogramming of murine somatic cells to induce pluripotent stem cells, which further highlights the significant involvement of this nuclear receptor in developmental gene networks [122, 123].

### 4.2. Steroidogenesis

The high expression levels of LRH‐1 observed in steroidogenic tissues, such as ovaries or testis, prompted further investigation into its biological role within these tissues. In breast cancer, LRH‐1 overexpression cooperates with estrogen receptor *α* (ER*α*) at estrogen‐response elements, increasing transcription of growth regulation by estrogen in breast cancer 1 (*Greb1*) and thyroid transcription factor‐1 (*Tff1*), and promoting tumor‐cell proliferation [100]. LRH‐1 can also enhance aromatase expression in breast adipose tissue, potentially contributing to increased local estrogen production and estrogen‐dependent tumor growth [101]. It has been shown that LRH‐1 regulates the expression of several key genes involved with steroidogenesis, such as *StAR* (steroidogenic acute regulatory protein), *CYP17* (17‐alpha‐hydroxyprogesterone aldolase), *HSD3B2* (3‐beta‐hydroxy‐delta(5)‐steroid dehydrogenase), and *CYP11B2* (aldosterone synthase) [102], where the rate‐limiting reaction in steroidogenesis is the conversion of cholesterol to pregnenolone, catalyzed by the enzyme cholesterol 7*α*‐hydroxylase (encoded by *CYP7A1*) [52]. Upon overexpression of LRH‐1, genes for cytochrome P450 A1 (*CYP11A1*) and B1 (*CYP11B1*) were also upregulated in isolated human pancreatic islets [124]. In the same study, the authors observed that LRH‐1 could protect islets against cytokine‐ or streptozotocin‐induced apoptosis [124]. These findings led to speculation that LRH‐1 mediated *β*‐cell protection against cytokines could, in part, be conveyed through the increased production of endogenous glucocorticoids. Moreover, it was shown that LRH‐1 regulates the expression of *CYP19*, the gene encoding aromatase, that is key to estrogen biosynthesis in breast adipose tissue [103, 125]. Endocrine resistance is a major challenge in the treatment of estrogen receptor‐positive breast cancer. In endocrine‐resistant breast cancer cells, NR5A2 was identified as a key transcriptional regulator of *HSD3B1*3 that encodes *β*‐hydroxysteroid dehydrogenase 1, which catalyzes conversion of dehydroepiandrosterone to androstenedione, a key precursor for estrogen biosynthesis [104].

The intestinal epithelium also produces glucocorticoids, so‐called “extra‐adrenal” synthesis [126]. In 2018, Huang et al. [127] discovered that synthesis of glucocorticoids is regulated by LRH‐1, and this correlated with immune responses in the intestinal mucosa. Their findings suggested that glucocorticoids may have important immunomodulatory activity to counter inflammation of the intestinal epithelium, and due to the regulation of glucocorticoids by LRH‐1, it is possible that LRH‐1 could be a potential therapeutic target for inflammatory bowel diseases [127]. Furthermore, the protective role of intestinal glucocorticoids against inflammation was shown to be mediated by LRH‐1 transcriptional activation of *CYP11A1* and *CYP11B1* genes [128]. Glucocorticoids can induce LRH‐1 expression via the GR, and LRH‐1 drives transcription of *SHP*, encoding the small heterodimer partner protein, which in turn acts as negative regulator of the pathway (Figure 2) [2]. It has been documented that SHP inhibits *β*‐catenin signaling in intestinal epithelial cells [129]. Based on these findings, it is possible that GR, LRH‐1, and SHP may form a regulatory network that modulates intestinal steroid production, although further studies that link all three transcription factors in this biological context are necessary.

**Figure 2:**
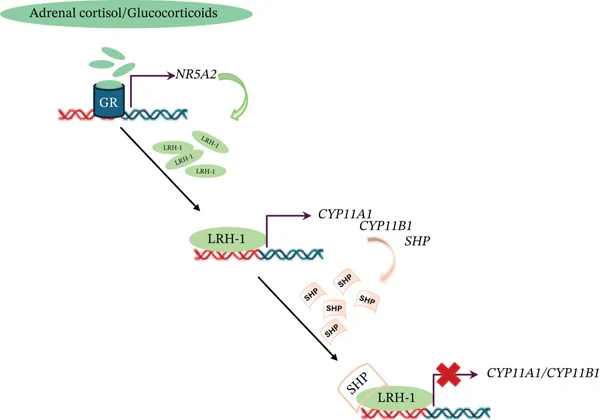
Potential regulatory network of intestinal glucocorticoid production. Glucocorticoid receptor (GR) activates *NR5A2* gene transcription and production of LRH‐1 upon binding cortisol or a glucocorticoid‐based drug. LRH‐1 triggers the expression of cytochrome P450 A1 (*CYP11A1*) and B1 (*CYP11B1*) genes, contributing to intestinal steroidogenesis. LRH‐1 also contributes to the expression of small heterodimer partner (SHP). SHP is a known negative regulator of LRH‐1‐dependent transcription, such as LRH‐1‐driven *CYP11A1* and *CYP11B1* transcription, leading to reduced intestinal steroid production.

### 4.3. Liver, Lipids, and Bile Acid Homeostasis

LRH‐1, highly expressed in liver, is implicated in regulating maturation of hepatocytes and in the maintenance of bile acids and cholesterol [130, 131]. It is also a key regulator of essential circadian metabolic genes in the liver, including protein phosphatase 1 regulatory subunit 3C (*Ppp1r3c*) involved in glycogen synthesis and storage, microsomal fatty acid elongase 5 (*Elovl5*) facilitating synthesis of long‐chain fatty acids, and *Cyp8b1* involved in bile acid synthesis [107]. The two key enzymes involved in bile acid biosynthesis, cholesterol 7*α*‐hydroxylase (encoded by *CYP7A1*) and sterol 12*α*‐hydroxylase (encoded by *CYP8B1*), are both regulated by LRH‐1 [108, 109]. LRH‐1 also controls the expression of scavenger receptor class B type 1 (SR‐B1) [105], a key receptor for uptake of HDL‐cholesterol in hepatocytes, with bile acids reducing its expression, also modulated by LRH‐1 [132]. Moreover, LRH‐1 regulates apical sodium‐dependent bile acid transporter (ASBT) and organic soluble transporters Ost*α*/*β* [110], to export bile acids and steroids from small intestine enterocytes [133]. SHP, also upregulated by bile acids, acts as a corepressor of LRH‐1, downregulating Ost*α*/*β*, suggesting an important negative feedback loop in enterocytes [110, 134]. Moreover, the *ABCB11* gene, encoding a key bile salt export pump (BSEP/ABCB11) that mediates the secretion of bile salts from hepatocytes into the bile, is also regulated by LRH‐1 [111, 135]. By regulating bile acid reabsorption and bile acid secretion genes, LRH‐1 is crucial to the enterohepatic circulation. LRH‐1 is also involved in lipid homeostasis in kidney podocytes, as shown recently both in vitro and in vivo using cultured podocytes and a diabetes‐induced mouse model of podocyte injury (lipotoxicity) [136].

In a murine model of liver cancer, LRH‐1 gain of function promotes mitochondrial glutamine metabolism by regulating glutaminase 2 (Gls2) and the glutamate pyruvate transaminase 2/glutamate oxaloacetate transaminase 1 (Gpt2/Got1) transaminase pathway, thereby promoting anaplerosis (replenishment of the intermediates of the citric acid cycle), mammalian target of rapamycin complex 1 (mTORC1) signaling, and HCC cell proliferation [112].

### 4.4. Glucose Homeostasis

LRH‐1 modulates glucose metabolism in skeletal muscle [113]. In C2C12 mouse myotube cultures, induction of LRH‐1 by DLPC induced mRNA levels of *Slc2a4*, *Hk2*, *Pfkm*, *and Pfkfb1* genes that encode glucose receptor, hexokinase II, phosphofructokinase, and 6‐phosphofructo‐2‐kinase/fructose‐2,6‐bisphosphatase 1, respectively. This led to decreased fatty acid degradation by modulating the activity of genes *Acc2*, *Cpt1b*, *Fabp3*, *Lpl*, *and Vldlr* that encode acetyl‐coenzyme A carboxylase beta, carnitine palmitoyltransferase 1b, fatty acid binding protein 3, lipoprotein lipase, and very low‐density lipoprotein receptor, respectively [113]. In the liver, LRH‐1 is a major regulator of *Gck* that encodes glucokinase, the enzyme responsible for the first step of glucose metabolism in host cells [114, 137].

### 4.5. Cell Renewal, Proliferation, and Transition

LRH‐1 modulates epithelial cell renewal and contributes to immune homeostasis via the induction of local glucocorticoid synthesis [5]. In the intestine, stem cells located at the base of the intestinal crypts, expressing the leucine‐rich repeat‐containing G‐protein coupled receptor 5 (LGR5), are responsible for rapid proliferation and intestinal regeneration [138]. Several key signaling pathways have been shown to impact epithelial cell proliferation and renewal: Wnt/*β*‐catenin [139], Notch [140, 141], and Hedgehog [142]. Paneth cells and mesenchymal cells secrete a plethora of key ligands, including Wnt ligands and Delta‐like 4 (DLL4) that activate the Wnt/*β*‐catenin and Notch signaling, respectively, and regulate intestinal stem cell proliferation [143]. Using cell lines and *Nr5a2* ^+/−^ mice, Botrugno et al. [80] showed that LRH‐1 and *β*‐catenin act synergistically downstream of the Wnt/*β*‐catenin pathway and drive intestinal stem cell renewal by regulating the transcription of cell cycle genes *CCND1* and *CCNE1* that encode cyclin D1 and cyclin E1, respectively. Deletion of *Nr5a2* in intestinal crypt stem cell‐derived organoids resulted in reduced proliferation of epithelial cells and significant changes in genes involved in cell survival and apoptosis [115]. Both *Notch1* transcripts and protein levels were diminished in Lrh‐1 intestinal enterocyte specific knockout (*Lrh-1^IEC-KO^*) mouse tissues with profound effects on the intestinal epithelial subpopulations [115]. Similarly, LRH‐1 is a major regulator of proliferation of granulosa cells within the ovarian follicle [144]. Moreover, LRH‐1 can be affected in response to other key signaling pathways such as Hedgehog, as seen in Zebrafish muscle [145]. In renal tubular epithelial cells, NR5A2 is seen to be an important regulator of epithelial‐mesenchymal cell transition (EMT) via transcriptional activation of *MMP25* encoding the zinc‐dependent matrix metallopeptidase 25 [116].

### 4.6. Pancreas Function

LRH‐1 is expressed in both the acinar and ductal cells of the exocrine pancreas [90]. LRH‐1 is important for the transcriptional regulation and expression of exocrine pancreas‐specific genes that encode lipases and proteases, secreted from acinar cells into pancreatic juice, involved in digestive breakdown of ingested food [118, 119]. RNA‐seq analysis and ChIP‐seq on whole pancreas revealed seven genes in which the LRH‐1 binding regulates their expression: *Pnlip* (pancreatic lipase), *Cel* (carboxyl ester lipase), *Ctrl* (chymotrypsin‐like), *Cpa1* (carboxypeptidase A1), *Ela1* (elastase 1), and transcription factors *Rbpjl* (recombination signal binding protein for immunoglobulin kappa J region‐like) and *Shp* [119].

## 5. LRH‐1 in the Immune System

Although the expression and functions of LRH‐1 in tissues of endodermal origin have been extensively studied, its involvement in the hematopoietic system remains considerably less explored. In 2011, Benod et al. [146] first reported the incidental detection of LRH‐1 expression in tumor‐infiltrating immune cells while they were investigating the role of this nuclear receptor in pancreatic cancer. Later, in 2015, direct evidence of the relevance of LRH‐1 in hematopoietic cells was provided by Lefevre et al. [147], who demonstrated the impact of LRH‐1 on macrophage polarization and since then, research has shifted towards further exploration of LRH‐1 within the immune system and its potential role in regulating immune cell functions.

### 5.1. Innate Immune System

The innate immune system acts as the body′s frontline defense against infections, featuring defense mechanisms primed for immediate action or rapidly triggered upon pathogen invasion. It includes both anatomic barriers, such as physical (epithelial layers of the skin and mucosa) and chemical (acidic pH, antimicrobial proteins, and peptides), as well as cellular responses [148]. A critical function of innate immunity is phagocytosis, an essential process for maintaining tissue health and eliminating infections [149]. The role of LRH‐1 remains largely unexplored in relation to innate immune cell types, such as neutrophils and dendritic cells; see Figure 3.

**Figure 3:**
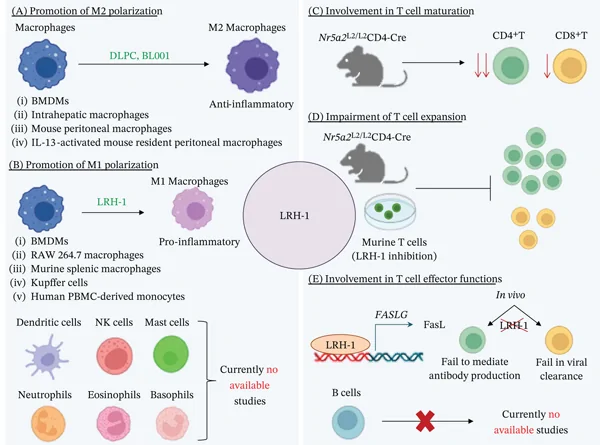
Direct relevance of LRH‐1 in the regulation and function of the immune system. Innate immune system: (*Α*) LRH‐1/NRA52 nuclear receptor ligand 1,2‐dilauroyl‐sn‐glycero‐3‐phosphocholine (DLPC) or synthetic LRH‐1 agonist BL001 can both effect a switch of macrophage polarization towards the M2 anti‐inflammatory phenotype, primarily affecting BMDMs, intrahepatic macrophages and peritoneal macrophages. (B) In contrast, LRH‐1 has some role in M1 macrophage polarization associated with proinflammatory responses, seen across various macrophage types (e.g. RAW 264.7 cell‐line, splenic macrophages, BMDMs, hepatic Kupffer cells, and human peripheral blood mononuclear cell [PBMC]–derived monocytes). Notably, the involvement of LRH‐1 in the biology of other innate immune cells (neutrophils, dendritic cells, natural killer [NK] cells, mast cells, basophils, and eosinophils) is yet to be explored. Adaptive immune system: (C–D) In vivo, T cell‐specific knockdown of LRH‐1 showcased its critical involvement in T cell maturation and expansion. *Nr5a2* ^L2/L2^ CD4‐Cre mice harboring this T‐cell‐specific deletion of LRH‐1, exhibited a prominent reduction of peripheral CD4^+^ T cells, whereas the impact of LRH‐1 deletion was less apparent regarding CD8^+^ T cell numbers. Assessment of the role of LRH‐1 in T cell expansion revealed impaired proliferation of LRH‐1‐deficient T cells, both in vivo and in vitro, with the CD4^+^ subset being more significantly impacted. (E) Evidence of the potential role LRH‐1 in T cell effector functions. LRH‐1 directly regulates the transcription of *FASLG*, encoding an essential T‐cell effector protein Fas/CD95 ligand (FasL). In vivo, LRH‐1 deletion blunted murine CD4^+^ T cell‐mediated antibody production, whereas LRH‐1 deficient CD8^+^ T cells failed to control virus expansion. Notably, the involvement of LRH‐1 in B lymphocyte biology is yet to be explored.

#### 5.1.1. Macrophages

LRH‐1 has been linked to the promotion of M2 polarization in macrophages. M2 macrophages, also referred to as alternatively activated macrophages, are associated with tissue repair, regeneration and the reduction of inflammation through the secretion of cytokines such as IL‐10 [150] and TGF‐*β* [151]. In contrast, M1 macrophages, or classically activated macrophages, are involved in proinflammatory responses, particularly in the initiation and maintenance of inflammation, and contribute to tissue damage [152]. In a key study utilizing the natural LRH‐1 ligand DLPC in a mouse model, Ghosh et al. [153] showed that the activation of LRH‐1 played a significant role in the switch of macrophage polarization towards the M2 anti‐inflammatory phenotype, primarily affecting bone marrow–derived macrophages (BMDMs) and intrahepatic macrophages, through the upregulation of signal transducer and activator of transcription 6 (STAT6) pathway. Similarly, Cobo‐Vuilleumier et al. [64] utilizing the synthetic agonist BL001 showed that LRH‐1 is an important modulator that promotes M2 macrophage polarization and enhances anti‐inflammatory cytokine production in mouse peritoneal macrophages. In line with these findings, Lefevre et al. [147] demonstrated that LRH‐1 promotes M2 polarization of macrophages in IL‐13‐activated mouse resident peritoneal macrophages, facilitating antifungal activity against *Candida* spp. Their experiments showed that LRH‐1 facilitates the anti‐inflammatory phenotype by synthesizing the peroxisome proliferator‐activated receptor gamma (PPAR*γ*) ligand (a molecule that binds to the PPAR*γ* nuclear receptor protein that regulates metabolism, adipogenesis, and inflammation) through a mechanism involving STAT6, which regulates the expression of two key enzymes, CYP1A1 and CYP1B1, essential for generating this ligand. Only one study to date, performed by Schwaderer et al. [154], has described that LRH‐1 is crucial for the activation of M1 macrophages, which are associated with proinflammatory responses. Pharmacological inhibition of LRH‐1 across various macrophage types demonstrated a marked decrease in the production of proinflammatory cytokines induced by bacterial lipopolysaccharide (LPS), although this study did not measure the levels of LRH‐1 (neither mRNA nor protein) under those experimental conditions [154].

Overall, it seems that LRH‐1 is expressed in macrophages and it is upregulated, both in vitro and in vivo, by specific agonists or during Th2 responses. Activation of this transcription factor leads to M2 macrophage polarization, with this macrophage subtype known to promote tissue repair and homeostasis and contribute to Th2 immunity, for example, in defense against *Candida albicans* infection. Inflammatory signals, such as LPS or IFN*γ*, lead to the downregulation of LRH‐1, which supports its role in tissue maintenance and not as a proinflammatory driver.

### 5.2. Adaptive Immune System

The adaptive immune system consists of a finely regulated interplay between lymphocytes and antigen‐presenting cells, which enable pathogen‐specific immunologic effector pathways and maintenance of host immune homeostasis. Over the last decade, a few studies have demonstrated a novel and direct role of LRH‐1 in adaptive immunity, specifically in T lymphocyte biology [120, 154–158]; see Figure 3.

#### 5.2.1. T‐Lymphocytes

Schwaderer et al. [154] were the first to provide evidence of LRH‐1 expression in murine primary and secondary lymphatic tissues as well as in murine mature T lymphocytes, although at much lower levels compared with murine liver and colonic tissue. A subsequent study revealed the low but detectable expression levels of *NR5A2* mRNA in human PBMCs [17]. The significantly lower expression levels compared with endodermal tissues do not, however, signify that LRH‐1 is less pertinent in T cell homeostasis and effector functions [17, 120]. To assess the potential role of LRH‐1 in the T cell lineage, Seitz et al. [17] generated a T cell‐specific LRH‐1/Nr5a2‐deficient murine model using CD4‐driven Cre recombinase (*Nr5a2* ^L2/L2^ CD4‐Cre, conditional knockout [cKO]). cKO mice harboring this T cell‐specific deletion of LRH‐1 exhibited a significant reduction in splenic size and weight, while no developmental or structural differences of the thymus were observed. On cellular level, the number of mature peripheral T cells in cKO mice was severely depleted and the absence of LRH‐1 was shown to have a differential impact on peripheral CD4^+^ versus CD8^+^ T cells. In cKO spleens, a threefold reduction of mature peripheral CD4^+^ T cell numbers was observed, whereas CD8^+^ T cells only decreased by half. This selective depletion of the peripheral T cell pool does not appear to result from increased sensitivity of cKO T cells to in vitro apoptotic stimuli, as their apoptotic response was reported to be comparable with that of control T cells. Instead, cKO CD4^+^ and, to a lesser extent, CD8^+^ T cells both underwent extensive cell death following impaired expansion in response to mitogenic stimulation, suggesting that the reduced peripheral T cell numbers were linked to a proliferative defect [14]. Either genetic knockout or specific pharmacological inhibition of LRH‐1 blunted activation‐induced proliferation of murine T cells in vitro, even though *Nr5a2*‐deficient T cells displayed signs of proper early activation (upregulation of CD25 and CD69). In vivo assessment of LRH‐1 effect in homeostatic expansion revealed extensive impairment of cKO CD4^+^ T cell expansion but only mild defects in the *Nr5a2*‐deficient CD8^+^ T cell population. The proliferative dependence on LRH‐1 was linked to its role in cell cycle regulation, as *Nr5a2* expression was rapidly induced upon TCR engagement, following the early induction of c‐Myc and preceding the induction of its transcriptional target, cyclin E1 [80]. Notably, the expression of both c‐Myc and cyclin E1 was noted to be reduced in activated LRH‐1‐deficient T cells, providing a likely molecular basis for the observed proliferation defect [14].

Beyond its role in T cell proliferation, LRH‐1 has also been implicated in the regulation of T cell effector functions. Specifically, LRH‐1 deletion inhibited murine CD4^+^ T cell effector function, including the ability to coordinate antibody production against model antigens in vivo and to induce intestinal inflammation in a transfer model of colitis [17]. Similarly, within the regulatory T cell (Treg) compartment, *Nr5a2*‐deficient Tregs failed to expand in vivo and consequently were unable to exert their protective capacity in a colitis transfer model, despite an unaffected baseline frequency of splenic FoxP3^+^ Tregs in cKO mice, suggesting that LRH‐1 is primarily required for Treg expansion rather than for maintaining their basal levels [14]. In the same study, during lymphocytic choriomeningitis virus (LCMV) infection of cKO mice, while cytotoxic CD8^+^ T cells exhibited standard activation, expansion and production of effector cytokines, they were unable to mediate viral clearance resulting in extensive tissue infection [17]. Additionally, LRH‐1 has been shown to influence T helper and cytotoxic T cell polarization, as genetic deletion of LRH‐1 in cKO mice enhanced Th1/Tc1‐associated cytokine responses, including IFN‐*γ* and IL‐2 secretion [17], whereas pharmacological activation of LRH‐1/*Nr5a2* in human PBMCs was associated with a shift towards Th2 responses, with reduced IFN‐*γ* and mildly increased IL‐4 secretion [159, 160].

The potential role of LRH‐1 in T cells, particularly in relation to their effector functions, was further investigated by Schwaderer et al. [120], where for the first time, LRH‐1 was shown to directly regulate the transcription of the crucial T‐cell effector molecule Fas/CD95 ligand (FasL/CD95L), a cell‐death promoting member of the tumor necrosis factor (TNF) family with critical roles in the regulation of T‐cell homeostasis and cytotoxicity [155]. The ligation of membrane‐bound FasL with its cognate receptor Fas (CD95) leads to the sequential activation of a caspase cascade that initiates apoptosis in target cells [156–158]. In human Jurkat T cells, LRH‐1 was found to directly regulate FasL expression via binding to specific sites in the *FASLG* promoter region. Specific pharmacological inhibition of LRH‐1 activity blunted FasL expression and reduced the FasL‐mediated cytotoxicity and cell death. In a murine model of concanavalin A‐induced and FasL‐dependent hepatitis, administration of LRH‐1 pharmacological inhibitors resulted in reduced hepatic FasL expression, coupled with a notable reduction of associated liver damage [120].

Taken together, these studies shed light on the fundamental role of LRH‐1/NR5A2 in T cell biology. Conventional CD4^+^ T cell peripheral maintenance, expansion and effector functions and CD8^+^ antiviral functions appear highly dependent on LRH‐1. Additionally, LRH‐1 is important for Th2‐type responses. Collectively, these findings position LRH‐1/NR5A2 as a promising therapeutic target in T‐cell–mediated pathologies, although its precise roles and underlying mechanisms across distinct T cell subsets remain to be fully elucidated. It is worth noting, however, that although LRH‐1 expression has been detected in B lymphocytes, its functional role in B cell biology remains unexplored.

## 6. Conclusions

LRH‐1/NR5A2 nuclear receptor has a broad spectrum of functions during embryogenesis and in adult life. LRH‐1 is shown to regulate early embryo development, determining stem cell pluripotency and somatic cell reprogramming. Its role in metabolism and cellular homeostasis has also been established in various experimental models and human tissue samples, especially liver, intestine, and pancreas. LRH‐1 is key in regulating cholesterol metabolism and steroidogenesis in tissues such as ovaries, testes, pancreas, and breast adipocytes, as well as driving glucocorticoid production in intestine, where their immunomodulatory activity is undoubtedly important in maintaining gut homeostasis and countering inflammation. Therefore, loss or reduced activity of LRH‐1 disrupts tissue‐specific regulatory mechanisms, contributing to inflammation, metabolic dysfunction, and disease progression. It is possible, therefore, that LRH‐1 could be a potential therapeutic target for chronic noncommunicable diseases, such as IBD and inflammation‐associated cancers. Similarly, LRH‐1 appears to be crucial to the enterohepatic circulation, regulating hepatic bile acid synthesis and secretion, and intestinal transport/reabsorption. Other key functions of this nuclear receptor include transcriptional regulation of genes involved in ethanol metabolism and glucose homeostasis. Surprisingly though, there is relatively poor knowledge of the role of LRH‐1 in the immune system, possibly due to the lower expression levels observed. Our review highlights fundamental roles for LRH‐1 in macrophage polarization and in T cell homeostasis, expansion and effector function. This nuclear receptor may therefore be a promising therapeutic target in treatment of immune cell mediated pathological disease. No studies to date have explored the association of LRH‐1 with respect to B lymphocyte biology. Further exploration is clearly needed, particularly given their role in mucosal immunity by producing specific antibodies for homeostasis, fighting pathogens, generating immunological memory, and acting as antigen‐presenting cells to help activate other immune cells crucial for the surveillance and protection of the body. The evolution of sequencing technologies and bioinformatic analytical approaches will no doubt further accelerate the identification of novel LRH‐1 immune‐specific functions and unlock deeper biological insights into LHR‐1 interaction networks within tissues, and with other organs, in health and disease.

## Author Contributions

B.J.C. and S.P.: conception and design. B.J.C., M.G., P.M., and S.P.: obtained funding. B.J.C., E.T., K.M., M.G., P.M., and S.P.: database search, acquisition, and review of articles. B.J.C. and S.P.: supervision of the project. B.J.C., E.T., K.M., M.G., P.M., and S.P.: drafted the manuscript.

## Funding

This study was supported by the State Scholarships Foundation (IKY), the British Embassy Athens, and the British Council Greece; the European Union Erasmus+ Traineeship Program; and a University of Thessaly Financial and Secretarial Administration of Research Grants Award (DEKA 5600.03.08.11).

## Disclosure

All authors critically appraised and approved the final submitted version of the manuscript.

## Conflicts of Interest

The authors declare no conflicts of interest.

## Data Availability

Data generated/analyzed during this study are included in this published article.
